# Acute Oxalate Nephropathy Associated with Orlistat: A Case Report with a Review of the Literature

**DOI:** 10.1155/2013/124604

**Published:** 2013-05-08

**Authors:** Dhara Chaudhari, Conchitina Crisostomo, Charles Ganote, George Youngberg

**Affiliations:** ^1^Department of Internal Medicine, East Tennessee State University, Quillen College of Medicine, Johnson City, TN 37615, USA; ^2^Department of Nephrology, East Tennessee State University, Quillen College of Medicine, Johnson City, TN 37615, USA; ^3^Department of Pathology, East Tennessee State University, Quillen College of Medicine, Johnson City, TN 37615, USA

## Abstract

Orlistat is a gastrointestinal lipase inhibitor used for weight reduction in obese individuals. Enteric hyperoxaluria caused by orlistat leads to oxalate absorption. Acute oxalate nephropathy is a rare complication of treatment with orlistat. Herein we report a patient presenting with acute renal failure which improved minimal with intravenous hydration. She was found to have oxalate crystals on renal biopsy. Patient admitted orlistat use over the counter for weight reduction on further questioning. The purpose of this case review is to increase awareness among patients since they are more focused on losing weight. This case also calls for the provider attention to educate patients regarding side effects of orlistat because of easy availability of orlistat over the counter.

## 1. Introduction

Orlistat, a gastrointestinal lipase inhibitor, is used for weight reduction in obese patients with BMI > 30 kg/m^2^ and BMI > 28 kg/m^2^ with associated risk factors such as diabetes mellitus hypertension. The majority of the side effects associated with orlistat involve gastrointestinal tract. The rare but serious adverse effect of orlistat treatment is acute oxalate nephropathy caused by increased fat malabsorption. It is diagnosed by evidence of oxalate crystals in renal biopsy specimen under polarized light. We report a case of obese patient consuming orlistat for weight reduction presented with acute oxalate nephropathy manifesting as acute renal failure.

## 2. Case

**Figure 1:**
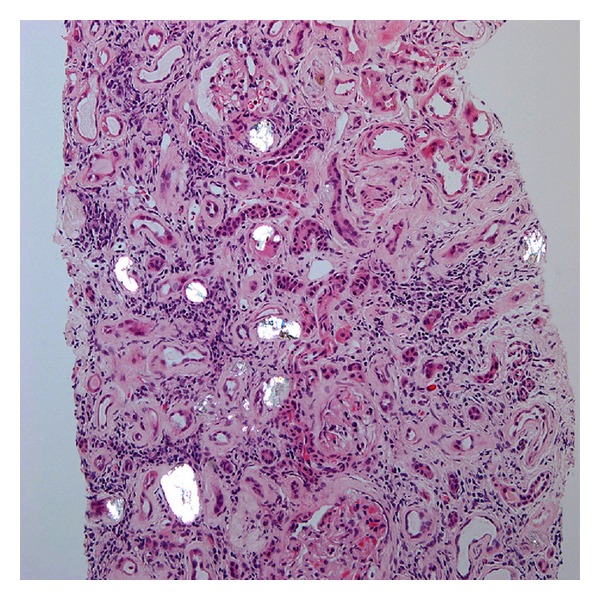
Hematoxylin and eosin stain of renal biopsy under polarized light (20x magnification) showing oxalate crystals in tubular lumen.

**Figure 2:**
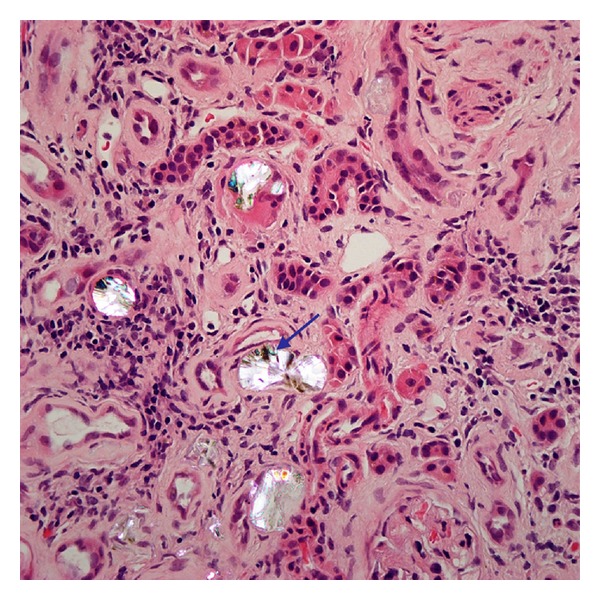
Hematoxylin and eosin stain of renal biopsy under polarized light (40x objective magnification) showing oxalate crystals with suggestion of “fan” like morphology (blue arrow).

A 56-year-old woman presented with fatigue to her primary doctor. She was sent to the hospital for acute kidney injury with a serum creatinine (Cr) of 6.6 mg/dL as compared to Cr of 0.9 mg/dL 1 year ago. The patient also had anemia with a hemoglobin of 7.4 g/dL. She denied having any previous medical problems but reported having intentionally lost 70 lbs over the last 18 months. She denied the use of any regular medications during her hospitalization and denied the use of nonsteroidal anti-inflammatory drugs (NSAIDs). Her physical examination was unremarkable. A urinalysis performed at admission was negative for urinary protein or eosinophils, with 2–5 WBCs per high powered field (hpf) and 0–2 RBCs/hpf, hemoglobin 7.4. The rest of complete blood count and basic metabolic panel was within normal limits. A renal ultrasound suggests medullary nephrocalcinosis. Her serum Cr improved with intravenous hydration to 5.7 mg/dL. She did not require hemodialysis and further clinical follow-up was performed on an outpatient basis. Her renal function failed to improve significantly over the next three months, which prompted a renal biopsy. The renal biopsy revealed severe interstitial fibrosis with associated tubular atrophy. On polarized light examination of the biopsy specimen, the renal pathologist detected moderate presence of oxalate crystals in tubular lumen (Figures 1 and 2). A 24-hour urine collection was analyzed and demonstrated normal oxalate levels and hypocitraturia. During a later follow-up appointment, the patient admitted to the use of orlistat. 

## 3. Discussion

Acute oxalate nephropathy is a form of kidney damage caused by oxalate crystal deposition in the renal tubular lumen. Acute oxalate nephropathy can be associated with primary or secondary hyperoxaluria. Oxalate is usually absorbed in the colon and oxalate absorption is limited by its binding to calcium forming insoluble calcium oxalate. There have been case reports of oxalate nephropathy attributed to the ingestion of high doses of vitamin C for a prolonged period [1]. Excessive ingestion of star fruit juice [2] and ethylene glycol [3] have also been implicated in oxalate nephropathy. Orlistat, a gastrointestinal and pancreatic lipase inhibitor, is a synthetic derivative of lipstatin used for weight reduction in patients with a BMI > 30 kg/m^2^. The beneficial effects of orlistat have extended beyond weight reduction to include an observed 37% reduction in the cumulative incidence of new onset diabetes mellitus [4]. Orlistat is associated with gastrointestinal side effects such as abdominal cramps, oily spotting, and fecal incontinence [5, 6]. The literature review revealed three cases associated with acute oxalate nephropathy with use of orlistat in obese patients [5, 7]. Proposed predisposing factors for acute oxalate nephropathy are dehydration, preexisting renal disease, and the simultaneous use of nephrotoxic drugs. The efficacy of orlistat is secondary to induction of fat malabsorption. The state of fat malabsorption created by orlistat can result in calcium binding to free fatty acids. Because of calcium and free fatty acids complex, a state of enteric hyperoxaluria is created which increases absorption of oxalate in the colon, thereby causing oxalate crystalluria and acute nephropathy. This mimics the mechanism of enteric hyperoxaluria in gastrointestinal conditions as can be found in Crohn's disease, postsmall bowel resection, and after intestinal bypass for morbid obesity [8]. A study done by Ferraz et al. on male rats after administration of Orlistat showed a 30% increase in urinary oxalate excretion [9]. Ferraz et al. hypothesized that Orlistat has the potential to increase the incidence of renal stone formation. Sarica et al. studied the effects of orlistat on intestinal oxalate absorption and urinary oxalate excretion in 95 overweight patients treated with Orlistat [10]. This study suggested that increased intestinal absorption contributes to urinary oxalate excretion which leads to renal stone formation. Karamadoukis et al. retrospectively reviewed 855 renal biopsies performed for tubular necrosis and crystals between 1997 and 2007. Two patients within the review were found to have acute tubular necrosis and calcium oxalate crystals; both patients were treated with orlistat [11]. In our case, even though 24-hour urine oxalate level was normal, renal biopsy under polarized light revealed oxalate crystals of moderate amount in tubular lumen. These oxalate crystals showed suggestion of “fan” like morphology on objective measurement of high power (40x) (Figure 2). The patient had reported using orlistat upon further questioning. Interventions that can potentially prevent a hyperoxaluric state include instituting a diet low in oxalate and calcium, increasing oral and intravenous fluid intake, and treatment with pyridoxine [7].
